# Molybdenum Complexes Derived from 2-Hydroxy-5-nitrobenzaldehyde and Benzhydrazide as Potential Oxidation Catalysts and Semiconductors

**DOI:** 10.3390/ijms25094859

**Published:** 2024-04-29

**Authors:** Jana Pisk, Mia Šušković, Edi Topić, Dominique Agustin, Nenad Judaš, Luka Pavić

**Affiliations:** 1 Department of Chemistry, Faculty of Science, University of Zagreb, Horvatovac 102a, 10000 Zagreb, Croatiaetopic@chem.pmf.hr (E.T.); judas@chem.pmf.hr (N.J.); 2 LCC-CNRS (Laboratoire de Chimie de Coordination), 205 Route de Narbonne, BP44099, CEDEX 4, 31077 Toulouse, France; dominique.agustin@iut-tlse3.fr; 3 Department of Chemistry, IUT Paul Sabatier, Université Paul Sabatier, University of Toulouse, Av. G. Pompidou, CS20258, 81104 Castres, France; 4 Division of Materials Chemistry, Ruđer Bošković Institute, Bijenička 54, 10000 Zagreb, Croatia; lpavic@irb.hr

**Keywords:** molybdenum, aroyl-hydrazones, electrical properties, oxidation, impendence spectroscopy, cyclooctene, linalool

## Abstract

This study aimed to synthesize molybdenum complexes coordinated with an aroyl hydrazone-type ligand (H_2_L), which was generated through the condensation of 2-hydroxy-5-nitrobenzaldehyde with benzhydrazide. The synthesis yielded two types of mononuclear complexes, specifically [MoO_2_(L)(MeOH)] and [MoO_2_(L)(H_2_O)], as well as a bipyridine-bridged dinuclear complex, [(MoO_2_(L))_2_(4,4’-bpy)]. Those entities were thoroughly characterized using a suite of analytical techniques, including attenuated total reflectance infrared spectroscopy (IR-ATR), elemental analysis (EA), thermogravimetric analysis (TGA), and single-crystal X-ray diffraction (SCXRD). Additionally, solid-state impedance spectroscopy (SS-IS) was employed to investigate the electrical properties of these complexes. The mononuclear complexes were tested as catalysts in the epoxidation of cyclooctene and the oxidation of linalool. Among these, the water-coordinated mononuclear complex, [MoO_2_(L)(H_2_O)], demonstrated superior electrical and catalytic properties. A novel contribution of this research lies in establishing a correlation between the electrical properties, structural features, and the catalytic efficiency of the complexes, marking this work as one of the pioneering studies in this area for molybdenum coordination complexes, to the best of our knowledge.

## 1. Introduction

Molybdenum is universally present and assumes a multifaceted function within environmental systems. It serves as an essential cofactor for various enzymes, while the dynamics of its interactions with organic molecules have been extensively explored. Research focusing on the synthesis and detailed examination of molybdenum complexes, utilizing diverse hydrazine ligands furnished with specific functional groups, constitutes a notable and intriguing domain within the field of coordination chemistry [1]. This line also significantly expands the potential practical applications of these materials. A review of the Cambridge Crystallographic Data Centre (CCDC) database reveals a limited number of entries for transition metal complexes incorporating aroylhydrazone derivatives of 2-hydroxy-5-nitrobenzaldehyde. Specifically, the database contains coordination complexes with V [2], Ni [3], Cu [4,5], Ru [6], Zn [7], and only one with Mo [8]. Certain Mo compounds exhibit properties such as antiviral, antitumoral, or antibiotic activity [9,10]. These coordination complexes are also recognized for their efficiency as catalysts in the oxidation of a wide array of organic substances, employing diverse oxygen donors such as peroxides, being ROOH and H_2_O_2_. Furthermore, dioxo and oxodiperoxo molybdenum(VI) species are frequently identified within the realm of oxidation catalysis and many molybdenum complexes serve as effective catalysts in the epoxidation of olefins, including nonfunctionalized olefins, using *tert*-butyl hydroperoxide (TBHP) as the oxygen donor [11,12,13]. TBHP can be commercially available in decane and water, the latter promoting the green chemistry principles. Among olefins, cyclooctene is frequently selected as a model substrate due to its reactivity and availability, which is the reason why cyclooctene is primarily tested in this study. Linalool, characterized by its double unsaturation and aliphatic alcohol functionality and existing in two isomeric forms of comparable stability, was selected as the secondary substrate. It is prevalent in numerous essential oils, notably those derived from Brazilian rosewood and lavender [14]. Despite its high market demand, natural sources alone are insufficient to meet the current needs for linalool. Consequently, the majority of linalool utilized commercially is synthesized from α-pinene, an abundant and cost-effective primary component of turpentine oils [15]. While linalool’s utilization in the pharmaceutical and fragrance sectors is well-documented, its use as a substrate in catalytic processes facilitates the synthesis of novel compounds. These compounds potentially exhibit enhanced stability, alongside novel aromatic profiles or functional properties, thereby expanding its application [16]. Palladium-catalyzed transformations of linalool were reported [17,18]. Utilizing methanol or ethanol as solvents, and employing catalytic quantities of [Pd(OAc)_2_] and [Cu(OAc)_2_], ethers of 7-hydroxyhotrienol (a naturally occurring component found in the oil of *Cinnamomum camphora*) were obtained. Further, an efficient synthetic pathway has been developed for the generation of linalool oxides, employing an environmentally friendly oxidizing agent, hydrogen peroxide (H_2_O_2_), in reactions catalyzed by the lacunary compound Na_7_PW_11_O_39_ at ambient temperature [19]. Through precise adjustment of reaction conditions, three valuable compounds were selectively synthesized. These include 2-(5-methyl-5-vinyltetrahydrofuran-2-yl)propan-2-ol and 2,2,6-trimethyl-6-vinyltetrahydro-2H-pyran-3-ol, both of which are linalool oxide derivatives. Additionally, a diepoxide derivative was successfully produced under these conditions. Moreover, a recent publication in this regard included a systematically conducted investigation about linalool oxidation employing [MoO_2_(SAP)]_2_ and [VO(SAP)]_2_O as catalysts, in the presence of environmentally benign oxidants TBHP (in water or decane) and H_2_O_2_ (with or without acetonitrile) [20].

In addition to the catalytic application of the prepared molybdenum coordination compounds, the key point of the presented study was their electrical investigation by impedance spectroscopy (IS) [21,22,23], as a new class of advanced materials. In that regard, the literature findings are quite poor and limited. For instance, published research has elucidated that Cu, Ni, and Co complexes of transition metals with Schiff bases exhibit promising capabilities for applications in electrochemical systems or as materials possessing nonlinear optical properties [24]. Additionally, vanadium complexes formed with tetradentate Schiff bases have been identified to display semiconducting characteristics, while metallic complexes of Cu(II), Co(II), and Hg(II) featuring the Schiff base *N*,*N*′-bis-(furaldehyde)-1,2-phenylenediamine-dipicolyl [25], as well as the *N*,*N*′-bis-(furaldehyde)-1,2-ethylenediamine-dipicolyl complex of Cu(II), when embedded within a plasticized PVC membrane, have been utilized in the fabrication of thiocyanate-selective electrodes with high selectivity [26]. Furthermore, vanadium coordination compounds derived from simple acyl-hzdrazones, containing 5-nitrobenzaldehyde, showed prominent potential as semiconducting materials [27]. Conversely, molybdenum-based complexes derived from closely related hydrazones as applied in this investigation, distinguished by the selection of hydrazide, specifically 2-hydroxybenzhydrazide (H_2_L^1*^ = Schiff base obtained by the reaction of 2-hydroxy-5-nitrobenzaldehyde and 2-hydroxybenzhydrazide) and 4-hydroxybenzhydrazide (H_2_L^2*^ = Schiff base obtained by the reaction of 2-hydroxy-5-nitrobenzaldehyde and 4-hydroxybenzhydrazide), exhibited enhanced electrical conductivity with rising temperature [28], which enables their classification as semiconductors. DC conductivity was determined: [MoO_2_(L^1*^)(MeOH)] to be 1.82 × 10^−9^, [MoO_2_(L^2*^)(MeOH)] 1.52 × 10^−14^, [MoO_2_(L^2*^)(H_2_O)] 3.43 × 10^−13^, and [MoO_2_(L^1*^)]*_n_* 3.43 × 10^−10^ (Ω cm)^−1^ @ 200 °C.

**Figure 1:**
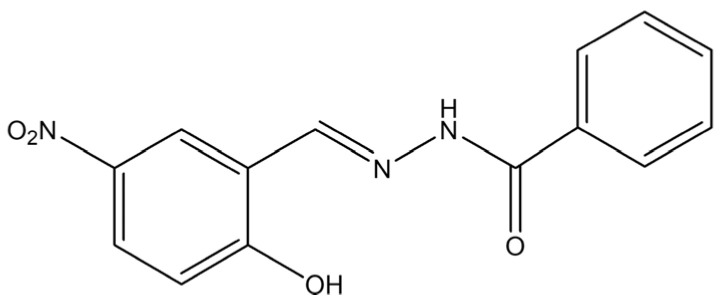
Ligand H_2_L used for the preparation of the Mo complexes.

In response to the recent literature contributions, the objective of this study was to synthesize molybdenum complexes coordinated by aroyl hydrazone-type ligands (Figure 1) and to conduct a comprehensive examination of their catalytic and electrical properties, providing materials with dual functionalities. The precursors used for the preparation of the molybdenum-containing materials belong to the category of cost-effective options, offering pragmatic economic solutions. To the best of our knowledge, this investigation represents the inaugural effort to establish a correlation between catalytic efficiency and electrical conductivity of those materials, thereby enhancing their potential application and value in various domains. The electrical properties of the synthesized compounds included the research of 4,4-bpyridine bridged Mo units, a class of materials previously unexplored in this context. On the other hand, the catalytic properties employed the use of linalool as a promising substrate.

## 2. Results and Discussion

### 2.1. Preparation, Spectroscopic Characterization, and Thermal Properties

The synthesized ligand (H_2_L) exhibits characteristic absorption bands in its infrared (IR) spectrum (SI, Figure S1). Bands observed at approximately 3304 cm^−1^ and 3071 cm^−1^ are attributed to the vibrations of the –OH and –NH bonds, respectively. Additionally, a significant absorption band is present at around 1601 cm^−1^, indicative of the C=N bond stretching of Schiff base structures. Furthermore, an absorption band at about 1633 cm^−1^ is associated with the stretching of the C=O bond. In the IR spectrum of the ligand, the nitro (NO_2_) group’s asymmetric and symmetric stretching vibrations are discernible, with wave numbers ranging from 1549 to 1482 cm^−1^ and from 1366 to 1291 cm^−1^, respectively. To prove the purity of the obtained ligand, DSC analysis was performed (SI, Figure S5). The DSC curve showed a pronounced, narrow peak indicative of the melting process. The onset of melting occurs at a temperature of 300 °C, with a melting enthalpy measured at 59 kJ/mol. To validate the synthesis of the ligand, its DSC curve was juxtaposed with that of benzhydrazide, facilitating the confirmation of the ligand’s formation.

Mononuclear molybdenum(VI) complexes were synthesized through the interaction of the ligand H_2_L with dioxobis(pentane-2,4-dionato)molybdenum(VI), denoted as [MoO_2_(acac)_2_], in methanol or acetonitrile. The interaction of H_2_L with [MoO_2_(acac)_2_] in methanol resulted in a yellow compound identified as [MoO_2_(L)(MeOH)] (**1**). In the IR spectra of the mononuclear complexes [MoO_2_(L)(MeOH)] (**1**) (SI, Figure S1), an absorption peak at 3498 cm^−1^ was detected, indicative of the O–H stretching vibration. Additionally, a characteristic band at 1603 cm^−1^ was observed, corresponding to the stretching vibrations of the C=N imine group. The presence of an vibration band at 1025 cm^−1^ suggests the coordination of methanol molecules, closely aligning with the typical MeOH vibration band at 1022 cm^−1^ [29,30]. These observations support the conclusion that the sixth coordination site on the central molybdenum atom in the [MoO_2_(L)(MeOH)] (**1**) complexes is occupied by a methanol molecule. Thermogravimetric analyses of molybdenum complex compounds were conducted in an oxygen atmosphere over a temperature range of 25 to 600 °C (SI, Figure S6), resulting in their conversion to MoO_3_ that was verified through the comparison of the infrared (IR) spectrum of the thermally derived residue with that of commercially available MoO_3_ (SI, Figure S4). For the [MoO_2_(L)(MeOH)] (**1**) complex, thermal decomposition also proceeded in two stages (SI, Figure S6, Table 1), with the first stage initiating at 143 °C and concluding at 167 °C. This stage exhibited a 6.6% mass loss, indicative of methanol (MeOH) release, compared to a theoretical value of 7.1%. The second stage, ranging from 331 °C to 442 °C, accounted for a 30.0% mass loss, correlating with a theoretical MoO_3_ yield of 32.3%. Further, the synthesis conducted in acetonitrile yielded yellow crystalline solids, the structure of which was elucidated as [MoO_2_(L)(H_2_O)] (**2**), based on the IR (SI, Figure S2) and thermogravimetric analysis (SI, Figure S7). For the complexes [MoO_2_(L)(H_2_O)] (**2**), an absorption band at 3466 cm^−1^ corresponds to O–H stretching vibrations, while the band at 1605 cm^−1^ is characteristic of C=N imine group stretching vibrations. Based on the yellow color of the complex, a coordinated solvent molecule could be assumed. Analysis of the IR spectrum of acetonitrile, provided a characteristic band at 2256 cm^−1^, corresponding to the vibrations of the C≡N bond, which was absent in the IR spectra of [MoO_2_(L)(H_2_O)] (**2**). For that reason, indicative O–H stretching vibrations suggested the occupancy of the sixth coordination site on the central molybdenum atom by a water molecule, which was additionally confirmed by thermogravimetry. The decomposition of [MoO_2_(L)(H_2_O)] (**2**) complex occurred in two distinct phases as well (SI, Figure S7, Table 1). The initial phase began at 143 °C and concluded at 167 °C, characterized by a 5.02% mass loss, aligning with the theoretical water loss of 4.20%. The subsequent phase, spanning 332 °C to 441 °C, resulted in a 31.5% mass loss, closely approximating the theoretical 33.2% expected for MoO_3_ formation. In both complexes, [MoO_2_(L)(MeOH)] (**1**) and [MoO_2_(L)(H_2_O)] (**2**), the *cis*-{MoO_2_}^2+^ core is evidenced by two strong absorption bands characteristic of Mo=O stretching vibrations, located in the spectral range from 920 to 910 cm^−1^ [29,30]. Upon conducting the aforementioned reactions in the presence of nitrogen bases such as pyridine, *γ*-picoline, or imidazole, it was observed that these bases did not participate in coordination to the molybdenum center. This observation was substantiated through comparative infrared (IR) spectroscopic analysis, indicating the specificity of ligand coordination to the metal center under the given reaction conditions.

Furthermore, a dinuclear complex [(MoO_2_(L))_2_(4,4-bpy)] (**3**) was isolated through the reaction of H_2_L and [MoO_2_(acac)_2_] in acetonitrile, with the addition of 4,4’-bpyridine (SI, Figure S3). In the IR spectra, the absorption band at 3100 cm^−1^ corresponds to N–H bond vibrations, while the band at 1599 cm^−1^ is indicative for C=N imine group stretching. The presence of bands from 1609 to 1540 cm^−1^ suggests the coordination of 4,4’-bpyridine, aligning with the typical absorption bands for the C=N group of pyridine found between 1615 to 1565 cm^−1^. Characteristic *cis*-{MoO_2_}^2+^ core absorption bands were found at 928 to 912 cm^−1^. In contrast to mononuclear complexes, the dinuclear complex [(MoO_2_(L))_2_(4,4-bpy)] (**3**) decomposed in a singular step, with decomposition temperatures ranging from 326 °C to 452 °C (SI, Figure S8, Table 1). This process led to a significant 71.2% mass loss, which, despite the apparent discrepancy, aligns with the theoretical MoO_3_ yield of 29.30%. The one-step decomposition phase highlights the distinct thermal behavior of dinuclear complexes compared to their mononuclear counterparts.

### 2.2. Description of Molecular and Crystal Structure

The crystal and molecular structures of [MoO_2_(L)(MeOH)] (**1**), [MoO_2_(L)(H_2_O)] (**2**), and [(MoO_2_(L))2(4,4-bpy)] (**3**) were determined by single-crystal X-ray diffraction (SCXRD) experiments. General and crystallographic data, as well as visualization and brief description of crystal structures, can be found in Supplementary Information, Tables S1–S3, and Figures S9–S11.

**Figure 2:**
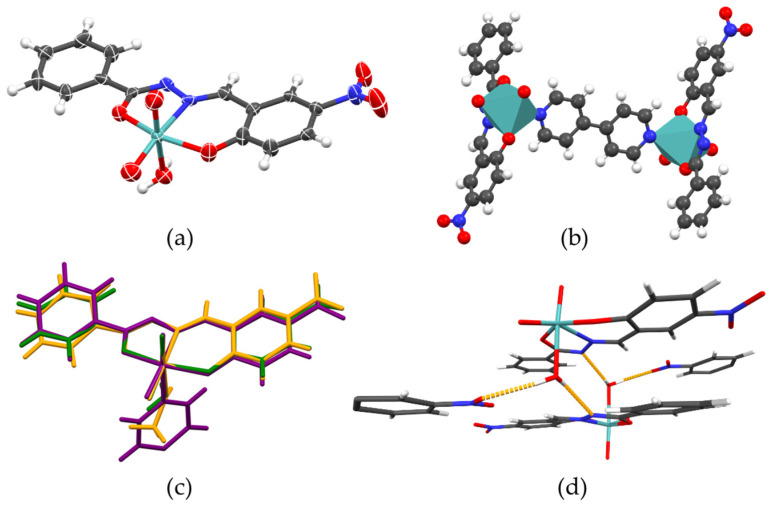
(**a**) ORTEP plot (50% probability level) of the representative molecular structure of the complex [MoO_2_(L)(H_2_O)] (**2**) and (**b**) polyhedral plot of the molecular structure of the dinuclear complex [(MoO_2_(L))_2_(4,4-bpy)] (**3**). All three complexes adopt similar coordination geometry, as shown in (**c**). Infinite supramolecular scaffolds are built only in [MoO_2_(L)(H_2_O)] (**2**) owing to a coordinated water molecule as ditopical hydrogen bond donor (**d**).

The complexes [MoO_2_(L)(MeOH)] (**1**), [MoO_2_(L)(H_2_O)] (**2**), and [(MoO_2_(L))2(4,4-bpy)] (**3**) share the same coordinative backbone well established for dioxomolybdenum(VI) complexes with ONO-donating ligands; see Figure 2a,c. According to the relevant bond lengths (SI, Table S2), the ligand conforms to enolato–imino–enolato tautomeric form regarding the hydrazonato and 2-oxoaryl moiety. The {MoO_2_}^2+^ core adopts distorted octahedral coordination with the sixth axial coordination site assuming significantly greater distance than the rest, as established in similar complexes with coordinated auxiliary ligands. Complex [(MoO_2_(L))2(4,4-bpy)] (**3**) built from ditopical coordination ligand 4,4’-bpy is, as expected, a dinuclear centrosymmetric complex, Figure 2b, similar to many previously synthesized [(MoO_2_(L))_2_(4,4-bpy)] complexes reported in the literature [31,32].

Even though the complexes have practically identical coordinative behavior, the packing of the complex molecules is dictated by the availability of the hydrogen bond donor sites (SI, Table S3). For the prepared complexes, the number of available hydrogen bond donors is two for [MoO_2_(L)(H_2_O)] (**2**), one for [MoO_2_(L)(MeOH)] (**1**), and none for complex [(MoO_2_(L))_2_(4,4-bpy)] (**3**). Consequently, no hydrogen bonds are observed in [(MoO_2_(L))2(4,4-bpy)] (**3**), while in [MoO_2_(L)(MeOH)] (**1**) supramolecular homodimers are bridged via the (methanol)O–H···N(imine) hydrogen bond. Finally, in [MoO_2_(L)(MeOH)] (**1**), with two available donor sites of the coordinated water molecule, the supramolecular dimers (equivalent to those found in [MoO_2_(L)(MeOH)] (**1**)) form supramolecular chains through (water)O–H···O(nitro) hydrogen bonds (Figure 2d).

### 2.3. Electrical Properties Investigation

Solid-state impedance spectroscopy (SS-IS) was employed to investigate the electrical properties of synthesized complexes. The temperature protocol for IS measurements was derived from TG analysis outcomes (referenced in Table 1), suggesting two cycles of thermal treatment ranging from room temperature (RT) to 200 °C, and cooling back to RT, with incremental steps of 10 °C. This specific maximum temperature threshold of 200 °C is justified by its capability to facilitate the removal of solvent molecules within the mononuclear Mo complexes [MoO_2_(L)(MeOH)] (**1**) and [MoO_2_(L)(H_2_O)] (**2**). The altered states of these complexes remain thermally stable up to approximately 320 °C. Conversely, complex [(MoO_2_(L))_2_(4,4-bpy)] (**3**) demonstrates an inherent thermal resilience, maintaining its structural composition without any observable transformations up to approximately 320 °C.

**Figure 3:**
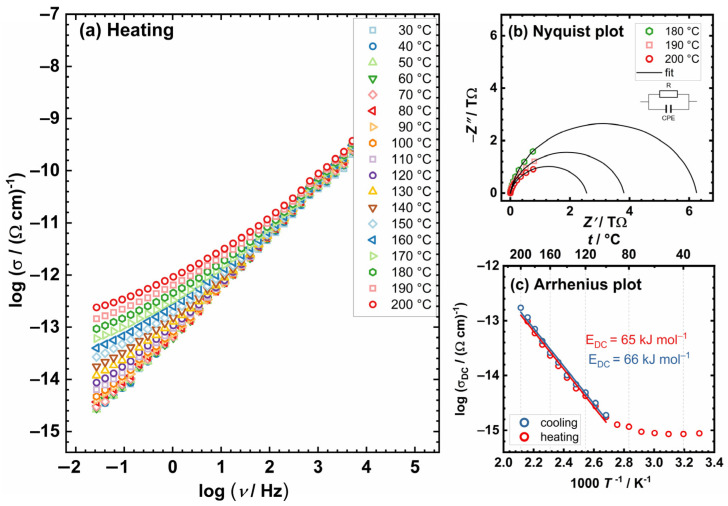
(**a**) Conductivity spectra; (**b**) Nyquist plot at different temperatures; and (**c**) Arrhenius plot—temperature dependence of DC conductivity (log(σ_DC_) vs. 1000/T) for complex [(MoO_2_(L))_2_(4,4-bpy)] (**3**). The corresponding equivalent circuit in (**b**) used for fitting the data is shown in the inset. Open squares denote experimental values; a solid black line corresponds to the best fit. In (**c**), the heating run is marked with red circles, and cooling is marked with blue circles. Solid lines in (**c**) represent the least-square linear fits to experimental data.

In light of the expected preservation of structural integrity throughout the thermal cycling process, complex [(MoO_2_(L))_2_(4,4-bpy)] (**3**) was prioritized for initial analysis. The conductivity spectra of complex [(MoO_2_(L))_2_(4,4-bpy)] (**3**), serving as a representative example for the entire series (after the first transformation of the complexes of studied complexes [MoO_2_(L)(MeOH)] (**1**) and [MoO_2_(L)(H_2_O)] (**2**)), are illustrated in Figure 3a. The frequency dependence of the real part of the electrical conductivity, *σ’*, at various temperatures reveals two distinct features. First, the spectra are dominated by frequency-dependent conductivity or conductivity dispersion (AC-part), particularly pronounced at lower temperatures and corresponding to the short-range transport of charge carriers. The second one, at the highest temperatures and lowest frequencies, begins with the formation of a frequency-independent region (DC-part) that corresponds to the DC conductivity that arises from the translational motions of charge. Similar features in conductivity spectra can be observed via IS in a wide range of semiconductive materials, crystalline and/or amorphous [27,28,33,34,35,36].

Owing to the lack of a discernible plateau in the graph, directly ascertaining the DC conductivity value from the plot is impractical. Therefore, this value is obtained by analyzing the experimental spectra of complex impedance. For all studied complexes, the impedance spectrum depicted in the complex impedance plane (commonly referred to as a *Nyquist plot*) contains a single semicircle associated with the bulk process, Figure 3b. This characteristic is indicative of electronic conductors; thus, a simplified EEC model (parallel R-CPE) is adopted for modelling, yielding corresponding fit parameters. The capacitance values obtained for the investigated process are found to be in the range of 10^−12^ F, and the parameter α exhibits values between 0.85 and 0.95. These observations correlate with the extent of semicircle depression observed in the impedance spectra and the deviation from ideal capacitive behavior. This suggests that the studied complexes exhibit a dual nature, manifesting properties characteristic of both resistors and capacitors. Resistance values, derived from the electrical equivalent circuit (EEC) analysis and the geometric parameters of the samples, were utilized to calculate the DC conductivity at various temperatures, as presented in Table 2. Specifically, for the complex [(MoO_2_(L))_2_(4,4-bpy)] (**3**), the DC conductivity was determined to be 5.9 × 10^−14^ (Ω cm) ^−1^ at 180 °C and 1.7 × 10^−14^ (Ω cm)^−1^ at 200 °C. The TGA analysis indicates that the complex [(MoO_2_(L))_2_(4,4-bpy)] (**3**) is anticipated to be stable within the studied temperature range, observed through TGA, and further supported by the in situ *IS* method. Observations indicate a consistent lack of variation between the heating and cooling cycles, implying that complex [(MoO_2_(L))_2_(4,4-bpy)] (**3**) maintains its structural integrity throughout, without undergoing any changes or transformations.

The DC conductivity is thermally activated and shows semiconducting behavior exhibiting Arrhenius temperature dependence and, hence, has characteristic activation energy; see Figure 3c. The activation energy for DC conductivity, *E*_DC_, was determined from the slope of log(*σ*_DC_) vs. 1000/T utilizing the following equation:

*σ*_DC_ = *σ*_0_*exp(−*E*_DC_/*k*_B_*T*), (1)

where *σ*_DC_ is DC conductivity, *σ*_0_* is the pre-exponential factor, *T* represents the temperature in K, and *k*_B_ is the Boltzmann constant. The investigated complexes exhibit a minimal temperature dependency, thereby necessitating the calculation of activation energy in the higher temperature domain (above 100 °C), where a linear relationship is observed. The values of *σ*_DC_ @200 °C and the activation energy *E*_DC_ values for all examined samples are summarized in Table 2 where the values obtained for the complexes [MoO_2_(L)(MeOH)] (**1**) and [MoO_2_(L)(H_2_O)] (**2**) are following analogue materials documented in reference [28]. Since the electrical properties of the bipyridine-bridged Mo units, present in the complex [(MoO_2_(L))_2_(4,4-bpy)] (**3**), to the best of our knowledge, has not been reported so far, this is the first example of conductivity and *E*_DC_ for this class of compounds.

**Figure 4:**
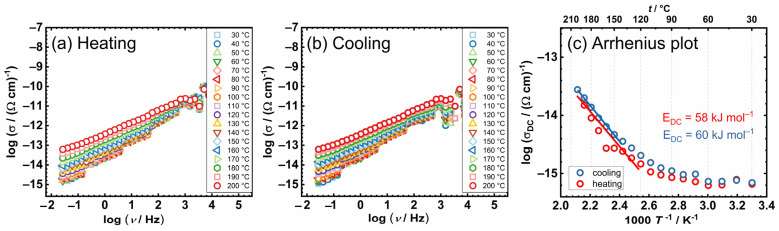
Conductivity spectra for [MoO_2_(L)(MeOH)] (**1**) in (**a**) heating and (**b**) cooling runs, and (**c**) Arrhenius plot—temperature dependence of DC conductivity (log(*σ*_DC_) vs. 1000/T) for both runs (red circle—heating, blue circle—cooling).

**Figure 5:**
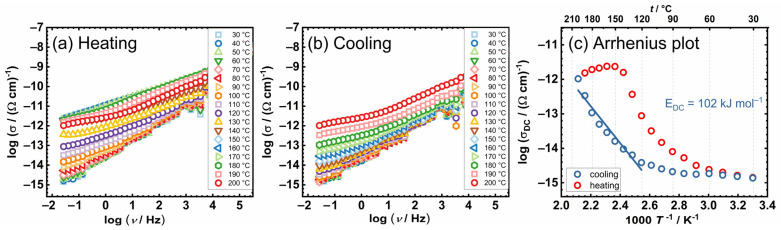
Conductivity spectra for [MoO_2_(L)(H_2_O)] (**2**) in (**a**) heating and (**b**) cooling runs, and (**c**) Arrhenius plot—temperature dependence of DC conductivity (log(*σ*_DC_) vs. 1000/T) for both runs (red circle—heating, blue circle—cooling).

Going forward, we conducted a detailed IS study on complexes [MoO_2_(L)(MeOH)] (**1**) and [MoO_2_(L)(H_2_O)] (**2**), both of which include coordinated solvents; Table 1. Conductivity spectra for these two complexes, encompassing both heating and cooling runs, along with Arrhenius trends are presented in Figure 4 and Figure 5.

As expected, the conductivity spectra display discernible variations during heating and cooling cycles, accompanied by non-monotonic changes in isotherms and DC conductivity, related to the release of coordinated solvent molecules through the heating cycle, as indicated by the thermal gravimetric (TG) analysis. For the sample [MoO_2_(L)(MeOH)] (**1**), this phenomenon is observed in situ as a non-monotonic alteration, specifically a noticeable inflexion in conductivity beyond 150 °C, with a pronounced deviation occurring between 160–170 °C, as illustrated in Figure 4a,c. This deviation corresponds to the initial decrement phase depicted in the TG curve. Conversely, during the cooling cycle, the DC conductivity exhibits a monotonic change. The release of the coordinated solvent molecule upon heating is anticipated to lead to the polymerization of the transformed complex into the [MoO_2_(L)]_n_ species.

Analysis of the Arrhenius trend of DC reveals that observed structural transformation and exit of methanol molecule slightly affect the conductivity. The activation energy was determined for both cycles, Table 2, showing similar values around ~60 kJ mol^−1^, which suggests that the transport mechanism does not change in the case of [MoO_2_(L)(MeOH)] (**1**) with structural transformation. Furthermore, in comparison to [(MoO_2_(L))_2_(4,4-bpy)] (**3**), [MoO_2_(L)(MeOH)] (**1**) shows almost one order of magnitude lower DC conductivity value (2.8 × 10^−14^ (Ω cm)^−1^ vs. 1.7 × 10^−13^ (Ω cm)^−1^ @200 °C) along with a similar range of *E*_DC_, which suggest slightly impeded electrical transfer for [MoO_2_(L)(MeOH)] (**1**).

Concerning [MoO_2_(L)(H_2_O)] (**2**), the release of the coordinated water molecule occurs at a slightly lower temperature, Table 1. This effect is similarly evident in in situ IS and is even more pronounced, as illustrated in Figure 5a–c. During the heating cycle of [MoO_2_(L)(H_2_O)] (**2**), in the first step of transformation, a notable sharp increase and non-monotonic change are observed with temperatures ranging 120–150 °C, Figure 5a. This is followed by a plateau in conductivity extending up to 200 °C, Figure 5c.

As the coordinated H_2_O (solvent) molecule departs from the complex [MoO_2_(L)(H_2_O)] (**2**), the aforementioned structural change strongly affects the electrical transport and IS conductivity spectra. It can be expected that the long parallel chains move closer as the structure is disturbed by heating. In the cooling cycle, we do not observe non-monotonic changes, indicating the stability of the transformed complex, which is expected to be polymerized [MoO_2_(L)]_n_ species. The activation energy determined during the cooling run is ~100 kJ mol^−1^, which is higher in comparison to transformed [MoO_2_(L)(MeOH)] (**1**). However, at the same time, DC conductivity is 1.0 × 10^−12^ (Ω cm)^−1^ @200 °C, which represents the highest value among all three complexes (1–3).

Observed trends are in line with the recent study of Sarjanovic et al. [28] on a similar class of compounds. In the case of transformed methanol- and water-coordinated complexes, as in our case complex [MoO_2_(L)(MeOH)] (**1**) and [MoO_2_(L)(H_2_O)] (**2**), it is expected to result in the same polymerized [MoO_2_(L)]_n_ species after heating, which is additionally indicated by the range of value for activation energy, around 60–65 kJ/mol. Furthermore, as in the previous study, *E*_DC_ for transformed H_2_O-coordinated complex [MoO_2_(L)(H_2_O)] (**2**) is higher (~100 kJ/mol). This effect could be related to the longer period needed for polymerization, and, as a result, it reflects upon the electrical properties and the activation energy. Moreover, it is interesting to stress that, as in Sarjanovic et al., complex [MoO_2_(L)(MeOH)] (**1**) shows low activation energy, however, its conductivity is two orders of magnitude (×100 times) lower compared to similar compounds where polymerization is expected upon heating, which we could relate to the type of ligand and slow release of the coordinated solvent during heating. All studied samples showed very similar DC values when compared to the same class of coordination complexes (water-coordinated and methanol-coordinated ones) with previously published ones [26].

### 2.4. Catalytic Investigation

**Figure 6:**
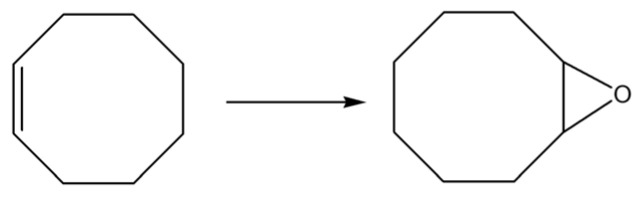
Cyclooctene (ep)oxidation pathway.

**Figure 7:**
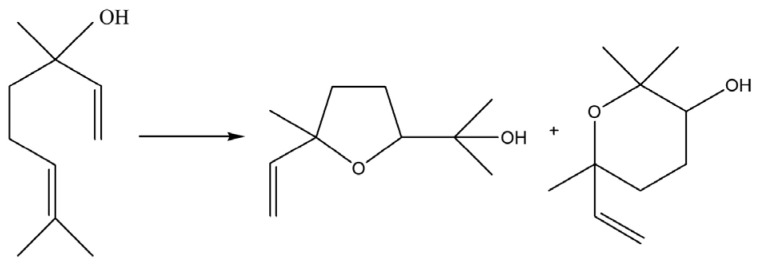
Linalool oxidation pathway.

**Figure 8:**
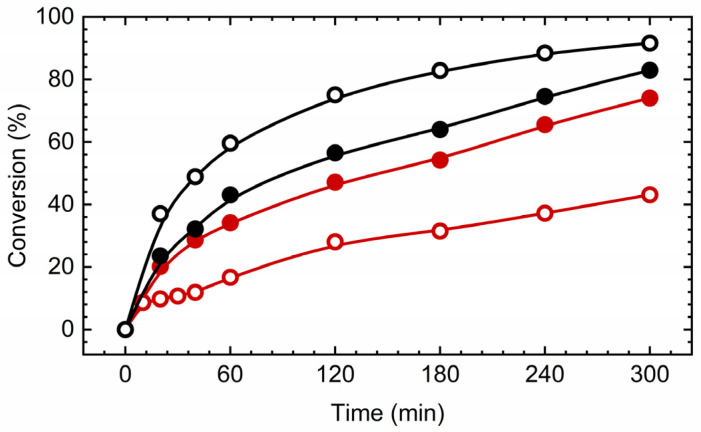
Kinetic profiles of substrate conversion with catalysts [MoO_2_(L)(MeOH)] (**1**), red line, and [MoO_2_(L)(H_2_O)] (**2**), black line. The full dot presents cyclooctene conversion, while the empty dot presents linalool conversion. The reaction temperature was 80 °C and TBHP in water was used as an oxidizing agent.

To examine the catalytic behavior of synthesized molybdenum (Mo) complexes, the study utilized mononuclear complexes [MoO_2_(L)(MeOH)] (**1**) and [MoO_2_(L)(H_2_O)] (**2**). The objective was to evaluate the catalytic efficiency of these complexes in oxidation reactions involving two distinct substrates: cyclooctene, serving as a reference substrate, and linalool. The expected and desired products of the oxidation reactions are presented in Figure 6 and Figure 7. *Tert*-butyl hydroperoxide (TBHP) in an aqueous solution was employed as the oxidizing agent, without the addition of any external solvents to the reaction mixture, preserving green chemistry principles. The obtained results are presented in Figure 8 and Table 3.

The kinetic analysis and data detailed in Table 3 reveal that the conversion efficiency of cyclooctene utilizing the catalysts [MoO_2_(L)(MeOH)] (**1**) and [MoO_2_(L)(H_2_O)] (**2**) achieved 73% and 83%, respectively. The selectivity towards the target epoxide was observed to be 65% for catalyst [MoO_2_(L)(MeOH)] (**1**) and 73% for catalyst [MoO_2_(L)(H_2_O)] (**2**). The turnover frequency (TOF) after 20 min was found to be within the range of 245–275 for both catalysts, with the turnover number (TON) spanning 300–320. It seems that the water-coordinated complex is slightly faster converted into pentacoordinated active species. Notably, the selectivity for the epoxide formation was higher with catalyst [MoO_2_(L)(H_2_O)] (**2**) (73%) compared to catalyst [MoO_2_(L)(MeOH)] (**1**) (65%). Given that cyclooctene served as the benchmark substrate, these outcomes are comparable to those obtained with analogous molybdenum-based catalysts previously reported in the literature. For instance, Mo complex coordinated by the ligand obtained by the reaction of pyridoxal and benzhydrazide, 0.25 mmol [MoO_2_(L)(MeOH)] [12], provided cyclooctene conversion of 56%, while the selectivity towards epoxide was 86%.

In addition, the catalytic performance observed with linalool as the substrate, in the presence of an aqueous oxidant, was notably promising. The conversion of linalool using catalyst [MoO_2_(L)(H_2_O)] (**2**) was 92%, significantly higher than the 43% conversion achieved with catalyst [MoO_2_(L)(MeOH)] (**1**), marking a reversal in the trend observed for cyclooctene oxidation. The selectivity towards the furanoid product was 41% for catalyst [MoO_2_(L)(MeOH)] (**1**) and 48% for catalyst [MoO_2_(L)(H_2_O)] (**2**), whereas selectivity towards the pyranoid product was 25% for catalyst [MoO_2_(L)(MeOH)] (**1**) and 29% for catalyst [MoO_2_(L)(H_2_O)] (**2**). The ratio of furanoid to pyranoid yields remained constant at 1.6 for both catalysts and in the trend observed in the literature. This comprehensive analysis underlines the nuanced efficiency and selectivity of these molybdenum-based catalysts in the oxidation of different substrates, demonstrating their potential versatility and applicability. In comparison, [MoO_2_(SAP)]_2_ catalyst, operating under the same conditions, Mo/Oxidant/L = 0.25/100/100, TBHP_aq_ as an oxidant, linalool conversion reached 90%, yield of 2-(5-methyl5-vinyl-tetrahydrofuran-2-yl propan-2-ol) 19% and 8% for 2,2,6-trimethyl-6-vinyl-tetrahydro-2H-pyran-3-ol, with F/P ration of 2.4. TOF_20 min_ was cca 500, while TON value reached 328.

## 3. Materials and Methods

### 3.1. Materials

Solvents and starting substances that are used as commercially available and without prior purification, and make them in order: ammonium paramolybdate (NH_4_)Mo_7_O_24_ · 4 H_2_O, (Kemika, Zagreb, Croatia), acetylacetone (C_5_H_8_O_2_, Kemika), nitric acid (HNO_3_, Sigma-Aldrich, Chemie, GmbH, München, Germany), 2-hydroxy-5-nitrobenzaldehyde (Sigma-Aldrich), benzhydrazide (Sigma-Aldrich), methanol (MeOH, Sigma-Aldrich), acetonitrile (MeCN, Sigma-Aldrich), pyridine, γ-picoline, 4,4-bpyridine (Aldrich Chemical), *cis*-cyclooctene, linalool (96%, TCI), 2-(5-methyl-5-vinyltetrahydro-furan-2-yl propan-2-ol) (97%, Aldrich), 2,2,6-trimethyl-6-vinyltetrahydropyran-3-ol (98%, TCI), TBHP (aqueous solution). Dioxobis(2,4-pentadionato)molybdenum(VI) [MoO_2_(acac)_2_] was prepared according to the literature procedure [37].

#### 3.1.1. Preparation of the Starting Compounds

**Synthesis of the H_2_L ligand**

In a 100 mL single-neck flask, 0.5 g (3.0 mmol) of 2-hydroxy-5-nitrobenzaldehyde was solubilized in 30 mL of methanol under heat until fully dissolved, forming a light-yellow solution. This solution was then subjected to reflux and maintained under heat for 20 min. Subsequently, 0.4073 g (3.0 mmol) of benzhydrazide was added to the flask. The addition of benzhydrazide resulted in the transformation of the solution’s color to orange, and this orange solution was further refluxed and heated for an additional three hours. Following the completion of the reaction, the orange, powdery product that formed was isolated via filtration. The yield of the filtered product was 0.7397 g, corresponding to an 86.4% yield.

IR-ATR: 3336 cm^−1^ (N–H), 1651 cm^−1^ (C=O), 1603 cm−1 (C=N), 1479 cm^−1^ (C=C), 1275 cm^−1^ (C–O).

#### 3.1.2. Synthesis of Molybdenum(VI) Complexes

**[MoO_2_(L)(MeOH)] (1)**

A ligand weighing 0.0500 g (0.1753 mmol) was placed into a single-neck flask and dissolved in 50 mL of methanol under heat to form a yellow solution. This solution was then subjected to reflux for 20 min. Subsequently, [MoO_2_(acac)_2_] (0.05752 g, 0.1753 mmol) was introduced into the yellow solution. The reaction mixture was stirred magnetically and maintained under reflux for three hours. Following this period, the yellow powdery product that had formed was isolated via filtration, yielding 0.0446 g of the complex, which corresponds to a 55.2% yield.

In a separate experiment, after dissolving the initial ligand in methanol and obtaining a solution, pyridine (C_5_H_5_N, 14,00 μL, 0.1753 mmol) was added. This modification resulted in the formation of the same complex with an increased yield of 0.0574 g (70.9%).

Furthermore, when *γ*-picoline (C_5_H_5_N, 17.1 μL, 0.1753 mmol) was used, a similar complex yield was achieved, with 0.0573 g being obtained (70.9% yield).

IR-ATR: 3498 cm^−1^ (–OH), 1603 cm^−1^ (C=N), 1025 cm^−1^ (MeOH), 920, 910 (Mo=O).

EA for C_15_H_13_MoN_3_O_7_: C_theo_: 40.56, C_found_:39.30, H_theo_: 2.96, H_found_: 3.01, N_theo_: 9.48, N_found_: 9.02%.

TG: MeOH_theo_: 7.2, MeOH_found_: 6.6, MoO_3 theo_: 32.30, MoO_3 found_: 30.06%.

**[MoO_2_(L)(H_2_O)] (2)**

The ligand (0.05000 g, 0.1753 mmol) was placed in a one-necked flask and dissolved in 30 mL of acetonitrile with heating. The resulting yellow solution was refluxed for 20 min. Into the resulting solution, [MoO_2_(acac)_2_] (0.05752 g, 0.1753 mmol) was added. The reaction mixture was stirred with a magnetic stirrer under reflux for three hours. The obtained yellow powder was filtered. A total of 0.03000 g of the complex was obtained (38.2% yield).

The same product was obtained when, after dissolving the ligand, pyridine was added (C_5_H_5_N, 14.14 μL, 0.1753 mmol). A total of 0.01890 g of the complex (24.0% yield) was obtained. With the addition of *γ*-picoline (C_5_H_5_N, 17.1 μL, 0.1753 mmol), 0.0421 g was obtained complex (53.6%).

IR-ATR: 3466 cm^−1^ (–OH), 1605 cm^−1^ (C=N), 922, 914 (Mo=O)

EA for C_14_H_11_MoN_3_O_7_: C_theo_: 39.18, C_found_: 38.76, H_theo_: 2.58, H_found_: 2.18, Nt_heo_: 9.79, N_found_: 9.04%.

TG: H_2_O_theo_: 4.2, H_2_O_found_: 5.02, MoO_3 theo_: 33.2, MoO_3 found_: 31.54%.

**[(MoO_2_(L))_2_(4,4-bpy)] (3)**

The ligand (0.05000 g, 0.1753 mmol) was placed in a one-necked flask and dissolved in 50 mL of acetonitrile with heating. The resulting light-yellow solution was refluxed for 20 min. Into the resulting solution, 4,4-bpyridine (0.02738 g, 0.1753 mmol) was added and after 10 min [MoO_2_(acac)_2_] (0.05752 g, 0.1753 mmol). The reaction mixture was mixed with a magnetic stirrer while refluxing for three hours. After three hours, the obtained orange powder product was filtered. The same product was obtained when half of the amount of ligand (0.025 g, 0.0876 mmol). A total of 0.0320 g of complex (74.6% yield) was obtained.

IR-ATR: 3100 cm^−1^ (N–H), 1599 cm^−1^ (C=N), 1609, 1540 cm^−1^ (4,4’-bpy), 928, 912 (Mo=O).

EA for C_38_H_26_Mo_2_N_8_O_12_: C_theo_: 46.45, C_found_: 46.12, Ht_heo_: 3.08, H_found_:3.45, N_theo_: 11.40, N_found_:10.78%.

TG: MoO_3 theo_: 29.30, MoO_3 found_: 28.80%.

#### 3.1.3. Catalytic Protocol

In a standard experimental setup, linalool or cyclooctene (20 mmol), along with an internal standard (0.1 mL) and catalyst (0.05 mmol), were combined in a round bottom flask. After regulating the reaction temperature to 80 °C, TBHP in water (40 mmol) was introduced to initiate the reaction. Over time, samples of the organic phase from the reaction mixture were periodically withdrawn and injected into GC for analysis.

To ensure the reproducibility of the obtained results all the measurements were performed twice.

### 3.2. Methods

Elemental analyses were conducted by the Analytical Services Laboratory of the Ruđer Bošković Institute, Zagreb.

IR-ATR infrared spectra were recorded using a Perkin Elmer 502 spectrophotometer (Perkin Elmer, Waltham, MA, USA) equipped with a diamond ATR accessory in the wavelength range numbers from 4400 to 450 cm^−1^. The spectra were processed and analyzed with the Omnic program.

Thermogravimetric analysis (TG) was performed using a Mettler-Toledo TGA/SDTA851 (Mettler-Toledo, Columbus, OH, USA) using aluminum oxide containers, under an oxygen atmosphere, and in a temperature range from 25 to 600 °C with a heating rate of 10 °C min^−1^. The obtained data were processed in the Mettler STARe program 16.01.

Differential scanning calorimetry (DSC) was performed using a Mettler-Toledo DSC823 calorimeter (Mettler-Toledo, Columbus, OH, USA) using aluminum vessels, under a nitrogen atmosphere, in the temperature range from 25 to 400 °C with a heating rate of 10 °C min^−1^. The obtained data were processed in the Mettler STARe program 16.01.

Single crystals of [MoO_2_(L)(MeOH)] (**1**), [MoO_2_(L)(H_2_O)] (**2**), and [(MoO_2_(L))2(4,4-bpy)] (**3**) of an appropriate quality were selected for the diffraction experiments. SCXRD experiments were performed using a Rigaku XtaLAB Synergy-S diffractometer (Rigaku, Tokyo, Japan) equipped with a Dualflex source (Cu *K*α radiation, *λ* = 1.54184 Å) and a HyPix detector. Intensities were collected via *ω*-scans at 170 K and data were processed using the CrysAlisPro v171.42.49 program package [38]. A summary of the general crystallographic data is presented in Table S1 (Supplementary Materials). The structures were solved by dual-space methods with SHELXT [39]. The refinement was performed via full-matrix least-squares methods based on *F*^2^ values against all reflections, including the anisotropic displacement parameters for all non-H atoms. Hydrogen atoms attached to carbon atoms were placed in geometrically idealized positions and refined by using the riding model, with *U*_iso_ = 1.2 *U*_eq_ of the connected carbon atom, or as ideal CH_3_ groups, with *U*_iso_ = 1.5 *U*_eq_. Hydrogen atoms attached to heteroatoms were located in the different Fourier maps in the final stages of the refinement procedure. All refinements were conducted using SHELXL [40] The SHELX programs were operated within the Olex2 suit [41]. Geometrical calculations were performed by Platon [42] and molecular graphics were produced using Mercury 2021.3.0 software [43]. CCDC 2341456-2341458 contains the supplementary crystallographic data for this paper. These data can be obtained free of charge from The Cambridge Crystallographic Data Centre via www.ccdc.cam.ac.uk/structures (accessed on 8 April 2024).

The electrical characteristics of complexes [MoO_2_(L)(MeOH)] (**1**), [MoO_2_(L)(H_2_O)] (**2**), and [(MoO_2_(L))2(4,4-bpy)] (**3**) were studied via solid-state impedance spectroscopy (SS-IS). Utilizing an impedance analyzer, specifically Novocontrol Alpha-AN Dielectric Spectrometer (Novocontrol Technologies GmbH & Co. KG, Hundsangen, Germany) impedance measurements were conducted over a wide range of frequencies (0.04 Hz to 1 MHz) and temperatures (30–200 °C, step 10 °C). The temperature was controlled to ±0.2 °C. The measurements were performed on powder polycrystalline samples pressed into pellets and gold electrodes were deposited on both sides of the disk using an SC7620 magnetron (Quorum Technologies, Laughton, UK) for electrical contact. The experimental data were analyzed using equivalent electrical circuit (EEC) modelling using the complex non-linear least-squares (CNLLSQ) fitting procedure and WinFit software software (Novocontrol Technologies GmbH & Co. KG, Hundsangen, Germany) [44]. For all studied complexes, the impedance spectrum contains a single semicircle. From the resistance values obtained from the fitting procedures, and the electrode dimensions (*d* sample thickness, and *A* electrode area) the DC conductivity is calculated. To ensure the reproducibility of the obtained results all the measurements were performed twice.

The catalytic reactions were followed by GC techniques on Agilent 6890A chromatograph (Agilent Technologies, Santa Clara, CA, USA) equipped with an FID detector and a DB5-MS capillary column (30 m × 0.32 mm × 0.25 mm). The GC parameters were quantified using authentic samples of the reactants and products. The conversion of cyclooctene and formation of the corresponding epoxide were calculated from calibration curves using authentic samples of the studied species relative to acetophenone as an internal standard (r^2^ = 0.998). The conversion of linalool and formation of 2-(5-methyl-5-vinyl-tetrahydrofuran-2-yl propan-2-ol) and 2,2,6-trimethyl-6-vinyltetrahydropyran-3-ol were calculated from calibration curves using authentic samples of the studied species relative to dodecane as an internal standard (r^2^ = 0.999).

## 4. Conclusions

In this study, we synthesized and characterized two mononuclear molybdenum complexes, [MoO_2_(L)(MeOH)] (**1**) and [MoO_2_(L)(H_2_O)] (**2**), along with a bpyridine-bridged dinuclear molybdenum(VI) coordination complex, [(MoO_2_(L))_2_(4,4-bpy)] (**3**). The coordinating ligand was derived from the condensation of 2-hydroxy-5-nitrobenzaldehyde and benzhydrazide. We utilized solid-state impedance spectroscopy to assess the electrical properties of these materials. Among them, the water-coordinated complex [MoO_2_(L)(H_2_O)] (**2**), in the cooling cycle, exhibited the highest DC conductivity, reaching 1.0 × 10^−12^ (Ω cm)^−1^ @200 °C. This enhanced conductivity could be attributed to the crystal packing of the mononuclear compounds, particularly the parallel chain layers observed in [MoO_2_(L)(H_2_O)] (**2**), which likely contribute positively to the electrical properties. The electrical properties of bipyridine bridged Mo compounds, complex [(MoO_2_(L))_2_(4,4-bpy)] (**3**), were examined for the first time, showing moderate DC conductivity and *E*_DC_. Additionally, the mononuclear complexes [MoO_2_(L)(MeOH)] (**1**) and [MoO_2_(L)(H_2_O)] (**2**) were tested as catalysts in the epoxidation of cyclooctene and the oxidation of linalool. The complex [MoO_2_(L)(H_2_O)] (**2**) demonstrated superior catalytic performance in terms of activity and selectivity towards the desired products. A novel aspect of this research is the correlation established between electrical properties, structural features, and catalytic performance, marking, to the best of our knowledge, one of the initial investigations of this kind within the realm of molybdenum coordination complexes, demonstrating the potential of multifunctional application.

## Figures and Tables

**Table 1:** TG analysis of the obtained Mo complexes.

Complex	Coordinated Solvent Loss/°C	Complex Decomposition/°C
[MoO_2_(L)(MeOH)] (**1**)	148–180	331–442
[MoO_2_(L)(H_2_O)] (**2**)	143–167	332–441
[(MoO_2_(L))_2_(4,4-bpy)] (**3**)	-	326–452

**Table 2:** Summary on IS data and electrical parameters of the obtained Mo complexes.

Complex	$\sigma_{DC}$/(Ω cm)^−1 a^	E_DC_/kJ mol^−1^ (Heating Run)	E_DC_/kJ mol^−1^ (Cooling Run)
[MoO_2_(L)(MeOH)] (**1**)	2.8 × 10^−14^	58.0	60.0
[MoO_2_(L)(H_2_O)] (**2**)	1.0 × 10^−12^	-	102.0
[(MoO_2_(L))_2_(4,4-bpy)] (**3**)	1.7 × 10^−13^	65.0	66.0

^a^ Measured @ 200 °C.

**Table 3:** Catalytic results of *cis*-cyclooctene and linalool oxidation reaction. Reaction conditions: time, 6 h; temperature, 80 °C, *n*(catalyst)/*n*(cyclooctene)/*n*(oxidant) = 0.05 mmol/20 mmol/40 mmol.

Substrate	Catalytic Parameter	Catalysts	
		[MoO_2_(L)(MeOH)] (1)	[MoO_2_(L)(H_2_O)] (2)
*Cis*-cyclooctene	Conversion ^a^/%	75	83
	Epoxide selectivity ^b^/%	65	73
	Epoxide yield/%	48	60
	TOF_20min_ ^c^	244	276
	TON ^d^	299	323
Linalool	Conversion ^a^/%	43	92
	Furanoid selectivity ^b^/%	41	48
	Furanoid yield/%	18	44
	Pyranoid selectivity ^b^/%	25	29
	Pyranoid yield/%	11	27
	TOF_20min_ ^c^	208	445
	TON ^d^	173	367

^a^ Substrate consumed at the end of the reaction. ^b^ Formed epoxide per converted olefin at the end of the reaction. ^c^ *n*(csubstrate)transformed/*n*(catalyst)/time(h) at 20 min. ^d^ *n*(substrate) transformed/*n*(catalyst) at the end of reaction.

## Data Availability

The data presented in this study are available from the corresponding author upon request.

## Supplementary Materials

The following supporting information can be downloaded at: https://www.mdpi.com/article/10.3390/ijms25094859/s1.
