# Supplementary material for: Molybdenum Complexes Derived from 2-Hydroxy-5-nitrobenzaldehyde and Benzhydrazide as Potential Oxidation Catalysts and Semiconductors
Source: Int J Mol Sci. 2024 Apr 29;25(9):4859. doi: 10.3390/ijms25094859 (PMC11084365; doi:10.3390/ijms25094859)
Supplement: Supplementary file 1 [file ijms-25-04859-s001.zip › ijms-2980646-supplementary.pdf]

Molybdenum complexes derived from 2-hydroxy-5-nitrobenzaldehyde and benzhydrazide as potential oxidation catalysts and semiconductors (Supporting Information)

Jana Pisk, Mia Šušković, Edi Topić, Dominique Agustin, Nenad Judaš and Luka Pavić

Table of Contents

Figure S1. IR spectra of [MoO_2(L)(MeOH)] (**1**) complex. 2

Figure S2. IR spectra of [MoO_2(L)(H_2O)] (**2**) complex. 2

Figure S3. IR spectra of [(MoO_2(L))$_2$(4,4-bpy)] **(3)** complex**.** 3

Figure S4. Comparison of IR spectra: residue after TG analysis (orange curve), commercially available MoO_3(black curve). 3

Figure S5. DSC curve for the ligand H_2L. 4

Figure S6. TG curve of [MoO_2(L)(MeOH)] (**1**) complex. 4

Figure S7. TG curve of [MoO_2(L)(H_2O)] (**2**) complex. 5

Figure S8. TG of [(MoO_2(L))$_2$(4,4-bpy)] (**3**) complex. 5

Table S1. Experimental and crystallographic data for complexes [MoO_2(L)(MeOH)] (**1**), [MoO_2(L)(H_2O)] (**2**) and [(MoO_2(L))$_2$(4,4-bpy)] (**3**). 6

Table S2. Selected bond lengths in the crystal structures of [MoO_2(L)(MeOH)] (**1**), [MoO_2(L)(H_2O)] (**2**) and [(MoO_2(L))$_2$(4,4-bpy)] (**3**). 7

Table S3. Hydrogen bond parameters in the crystal structures of prepared complexes [MoO_2(L)(MeOH)] (**1**), [MoO_2(L)(H_2O)] (**2**) and [(MoO_2(L))$_2$(4,4-bpy)] (**3**). 8

Figure S9. (a) Molecular structure of [MoO_2(L)(MeOH)] (**1**). Atoms are shown as thermal ellipsoid with 50% probability level. The asymmetric unit contains one whole molecule of the complex. (b) Packing of mononuclear complexes [MoO_2(L)(MeOH)] (**1**) in the crystal structure. The molecules form supramolecular $R22(10)$ homodimers formed by methanol O–H···N1 imido hydrogen bonds. No other significant interactions between the dimers are observed. 9

Figure S10. (a) Molecular structure of [MoO_2(L)(H_2O)] (**2**). Atoms are shown as thermal ellipsoid with a 50% probability level. The asymmetric unit contains one whole molecule of the complex. (b) Packing of mononuclear complexes [MoO_2(L)(H_2O)] (**2**) in the crystal structure. The molecules form supramolecular $C44(26)$ chains spanning through water O–H···O6(nitro) hydrogen bonds and water O–H···N1 imido hydrogen bond. The latter enables the formation of supramolecular homodimers connected via hydrogen bonds with nitro moiety. (c) Packing of mononuclear complexes **2** in the crystal structure. Molecules are colored by symmetry operation, showing the formation of supramolecular dimers bridged by O–H···O6(nitro) hydrogen bonds. 11

Figure S11. (a) Molecular structure of [(MoO_2(L))$_2$(4,4-bpy)] (**3**). Atoms are shown as thermal ellipsoid with a 50% probability level. The asymmetric unit contains the upper half of the shown molecule only (center of inversion is situated at the midpoint of 4,4'-bpy C–C bond). (b) Packing of dinuclear complexes [(MoO_2(L))$_2$(4,4-bpy)] (**3**) in the crystal structure. The molecules do not form any significant intermolecular interactions due to the lack of hydrogen bond donors. 12

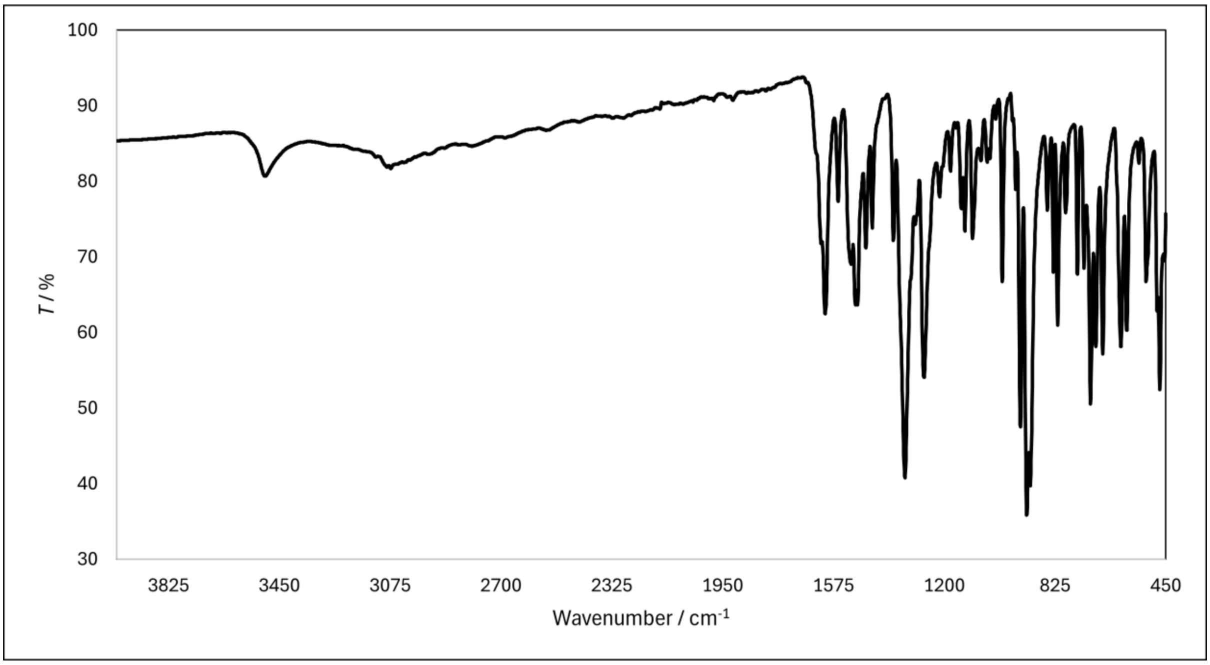

Figure S1. IR spectra of [MoO_2(L)(MeOH)] (**1**) complex.

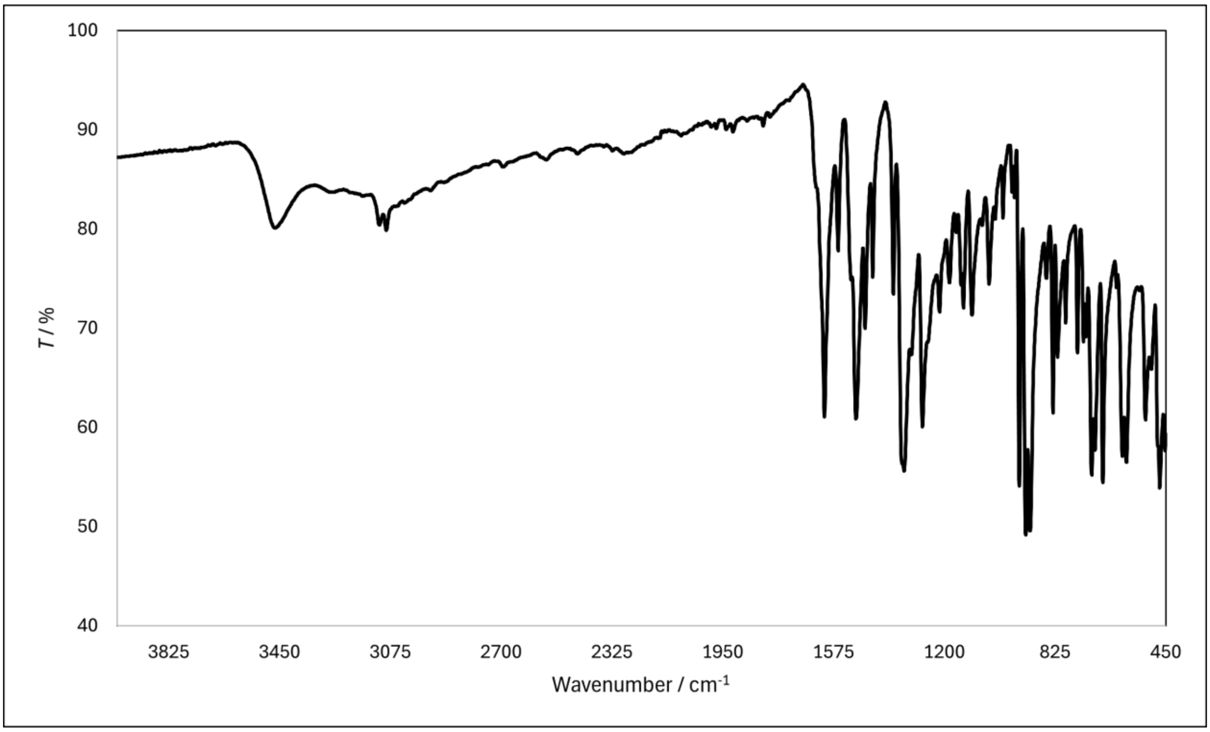

Figure S2. IR spectra of [MoO_2(L)(H_2O)] (**2**) complex.

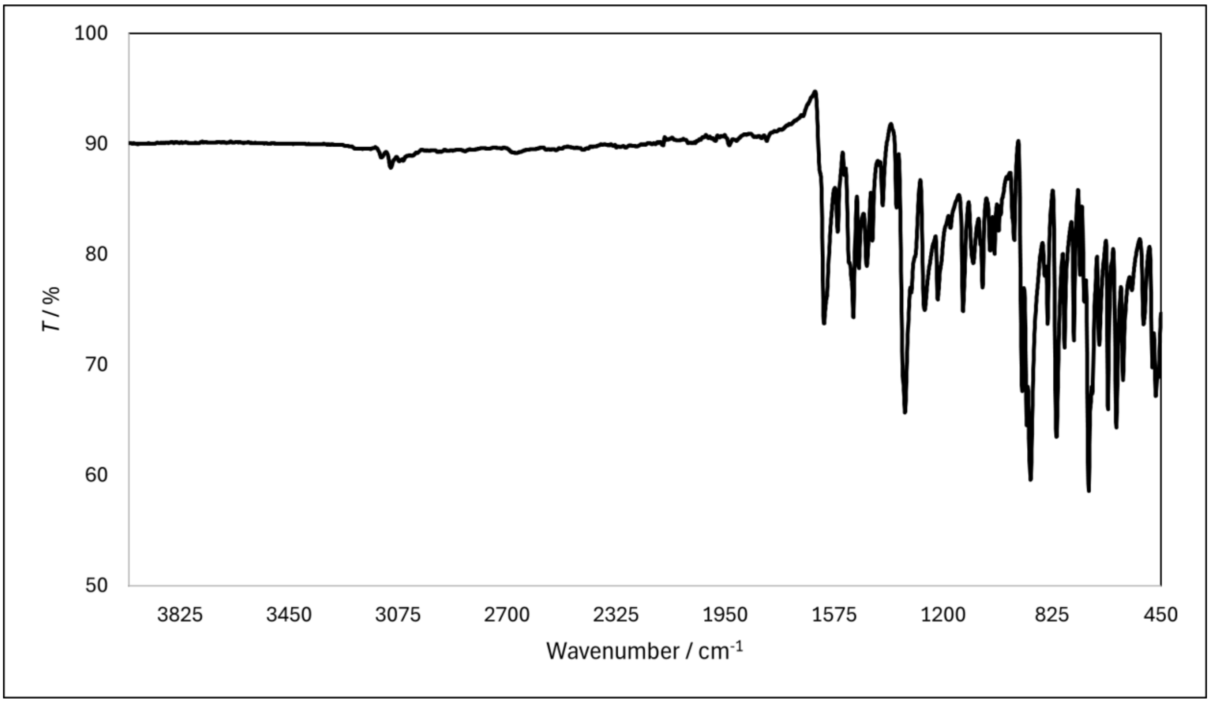

Figure S3. IR spectra of [(MoO_2(L))$_2$(4,4-bpy)] (**3**) complex.

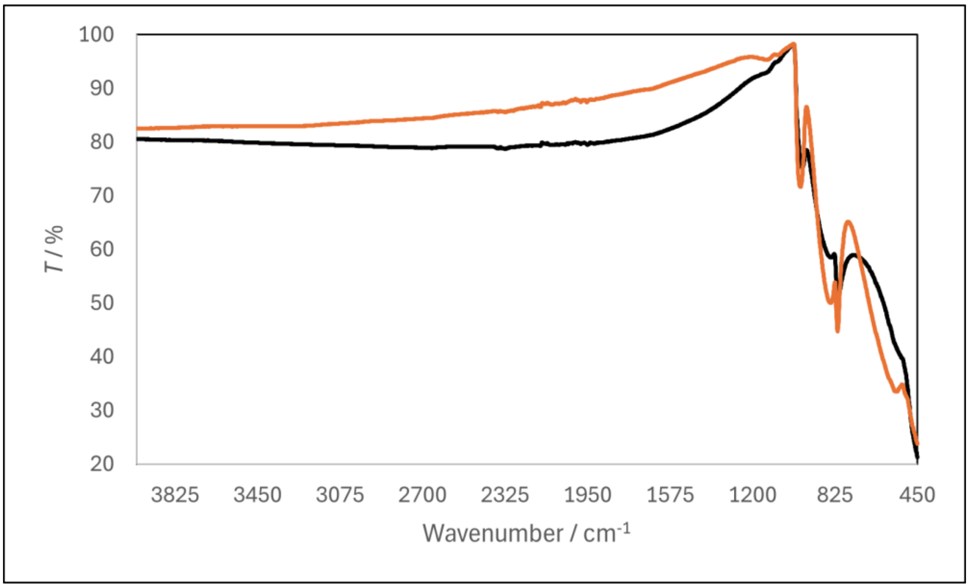

Figure S4. Comparison of IR spectra: residue after TG analysis (orange curve), commercially available MoO_3(black curve).

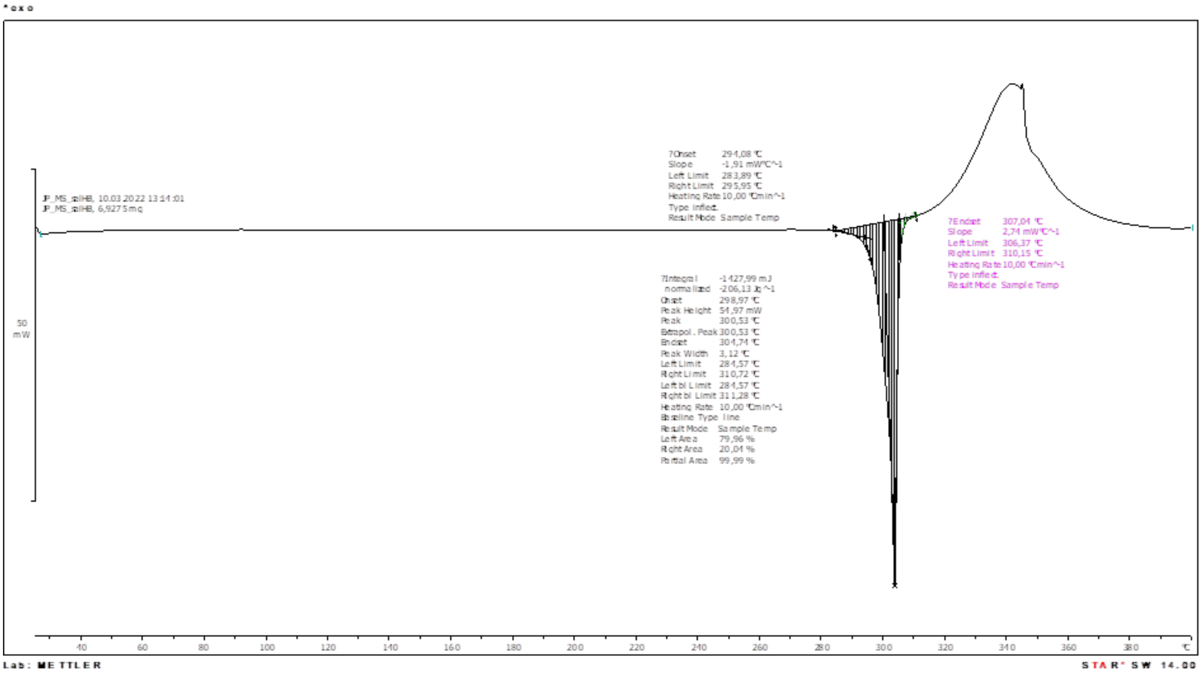

Figure S5. DSC curve for the ligand H_2L.

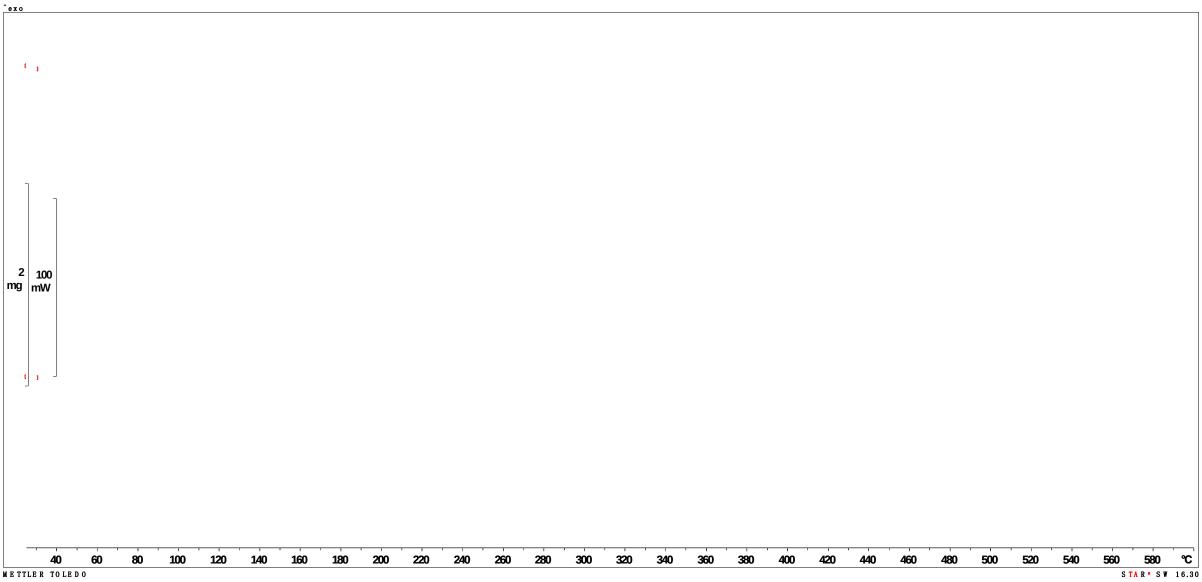

Figure S6. TG curve of $[MoO_2(L)(MeOH)]$ (**1**) complex.

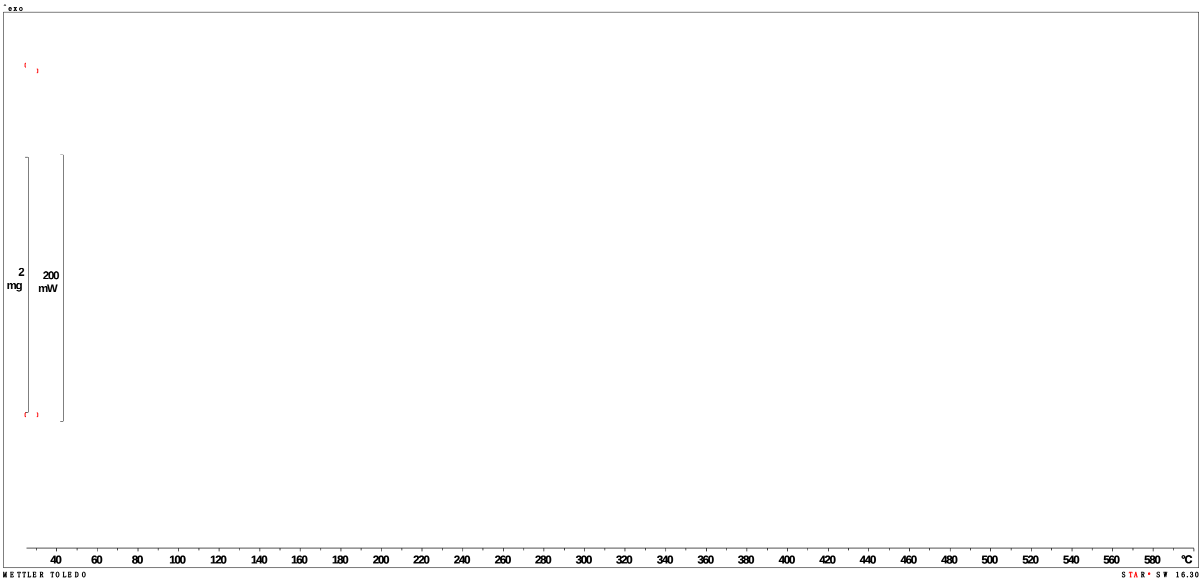

Figure S7. TG curve of $[MoO_2(L)(H_2O)]$ (**2**) complex.

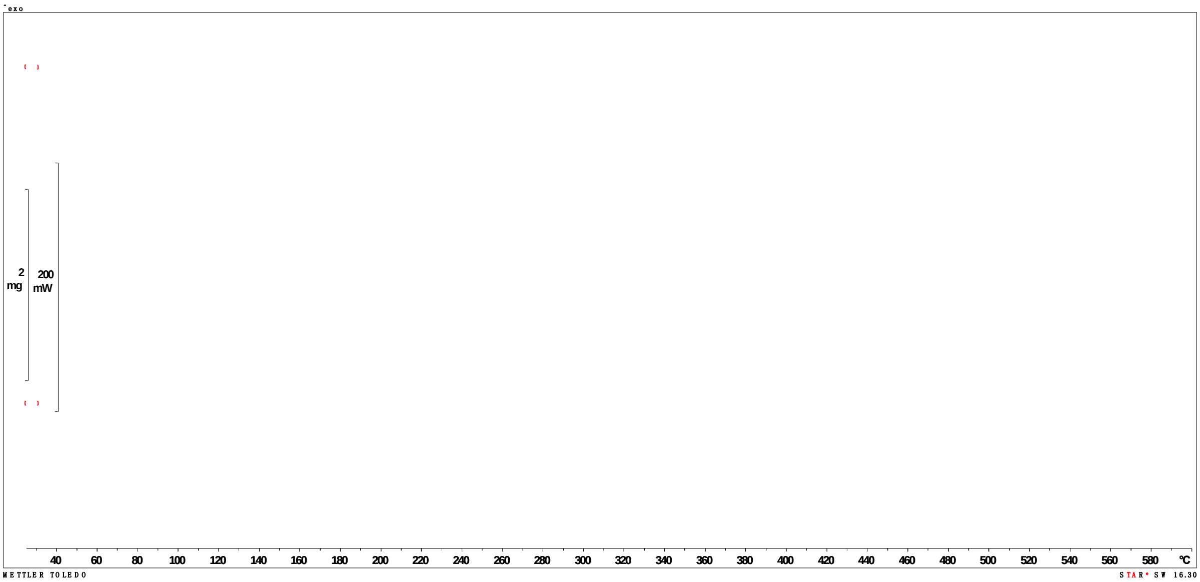

Figure S8. TG of $[(MoO_2(L))_2(4,4\text{-bpy})]$ (**3**) complex.

Table S1. Experimental and crystallographic data for complexes [MoO_2(L)(MeOH)] (**1**), [MoO_2(L)(H_2O)] (**2**) and [(MoO_2(L))$_2$(4,4-bpy)] (**3**).

Identifier	[MoO_2(L)(MeOH)] (**1**)	[MoO_2(L)(H_2O)] (**2**)	[(MoO_2(L))$_2$(4,4-bpy)] (**3**)
Empirical formula	$C_{15}H_{13}MoN_3O_7$	$C_{14}H_{11}MoN_3O_7$	$C_{38}H_{26}Mo_2N_8O_{12}$
M_r	443.22	429.20	978.55
T/K	170(2)	169.98(10)	169.99(10)
Crystal system	triclinic, yellow needle	monoclinic, yellow plate	monoclinic, yellow plate
Space group	P –1	P 1 $2_1/c$ 1	P 1 $2_1/n$ 1
a/Å	7.9589(3)	10.8039(7)	13.1888(4)
b/Å	9.5589(3)	10.7689(6)	10.5504(3)
c/Å	10.5117(3)	13.5832(8)	14.1743(4)
α/°	83.639(2)	90	90
β/°	88.860(3)	108.087(7)	106.292(3)
γ/°	89.126(3)	90	90
V/Å^3	794.56(4)	1502.26(17)	1893.11(10)
Z	2	4	2
ρ_{calc}/g cm^{-3}	1.853	1.898	1.717
μ/mm^{-1}	7.200	7.592	6.092
F(000)	444	856	980
Crystal size/mm^3	0.08×0.03×0.03	0.08×0.05×0.004	0.3×0.08×0.02
Radiation			Cu Kα (λ = 1.54184Å)
2Θ range/°	8.466 to 155.052	8.61 to 154.716	8.096 to 155.81
Index ranges	$-10 \le h \le 10$, $-12 \le k \le 9$, $-13 \le l \le 13$	$-13 \le h \le 12$, $-13 \le k \le 13$, $-16 \le l \le 15$	$-16 \le h \le 16$, $-13 \le k \le 13$, $-16 \le l \le 17$
Reflections collected	12446	10107	21369
Independent reflections	3268 [R_{int} = 3.58%, R_{sigma} = 4.08 %]	3007 [R_{int} = 6.13%, R_{sigma} = 6.47 %]	4006 [R_{int} = 6.9%, R_{sigma} = 10.16 %]
Data/restraints/parameters	3268/-/239	3007/-/227	4006/-/271
g_1, g_2 in w^a	0.0579, 1.3589	0.0531, 46.7310	0.1059, 0
Goodness–of–fit on F^2, S^b	1.079	1.057	1.048
Final R and wR^c values [$I \ge 2\sigma(I)$]	R_1 = 3.62%, wR_2 = 9.81%	R_1 = 8.67%, wR_2 = 21.35%	R_1 = 5.19%, wR_2 = 14.31%
Final R and wR^c values [all data]	R_1 = 3.86%, wR_2 = 9.98%	R_1 = 11.31%, wR_2 = 22.69%	R_1 = 5.71%, wR_2 = 15.02%
Largest diff. peak/hole / e Å^{-3}	1.076/–1.187	3.097/–1.384	0.873/–1.330

[a] $w = 1/[\sigma F_o^2 + (g_1P)^2 + g_2P]$ where $P = (F_o^2 + 2F_c^2)/3$

[b] $S = \{\Sigma[w(F_o^2 - F_c^2)2]/(N_r - N_p)\}^{1/2}$ where N_r = number of independent reflections, N_p = number of refined parameters.

[c] $R = \Sigma||F_o| - |F_c|/\Sigma|F_o|$; $wR = \{\Sigma[w(F_o^2 - F_c^2)2]/\Sigma[w(F_o^2)2]\}^{1/2}$

Table S2. Selected bond lengths in the crystal structures of [MoO_2(L)(MeOH)] (**1**), [MoO_2(L)(H_2O)] (**2**) and [(MoO_2(L))$_2$(4,4-bpy)] (**3**).

[MoO_2(L)(MeOH)] (**1**)					
Atoms	Bond length/Å	Atoms	Bond length/Å	Atoms	Bond length/Å
C2–C3	1.390(5)	C12–C13	1.392(5)	N1–N2	1.400(4)
C2–C7	1.396(4)	Mo1–N2	2.260(3)	O1–C1	1.312(4)
C3–C4	1.391(5)	Mo1–O1	2.007(2)	O2–C10	1.326(4)
C4–C5	1.385(5)	Mo1–O2	1.939(2)	O5–C15	1.423(6)
C9–C14	1.394(4)	Mo1–O3	1.696(3)	O6–N3	1.221(4)
C10–C11	1.409(5)	Mo1–O4	1.702(3)	O7–N3	1.223(4)
C11–C12	1.371(6)	Mo1–O5	2.309(2)		
[MoO_2(L)(H_2O)] (**2**)					
Atoms	Bond length/Å	Atoms	Bond length/Å	Atoms	Bond length/Å
C2–C7	1.382(17)	C13–C14	1.385(16)	N1–C1	1.271(16)
C3–C4	1.40(2)	Mo1–N2	2.234(10)	N1–N2	1.416(12)
C4–C5	1.37(2)	Mo1–O1	2.021(8)	O1–C1	1.316(14)
C5–C6	1.38(2)	Mo1–O2	1.940(10)	O2–C10	1.330(17)
C10–C11	1.411(17)	Mo1–O3	1.742(12)	O6–N3	1.233(14)
C11–C12	1.380(19)	Mo1–O4	1.690(11)	O7–N3	1.210(15)
C12–C13	1.375(17)	Mo1–O5	2.270(10)		
[(MoO_2(L))$_2$(4,4-bpy)] (**3**)					
Atoms	Bond length/Å	Atoms	Bond length/Å	Atoms	Bond length/Å
C9–C10	1.414(5)	Mo1–N4	2.395(3)	N4–C15	1.343(5)
C9–C14	1.392(6)	Mo1–O1	1.995(2)	N4–C19	1.339(5)
C10–C11	1.396(6)	Mo1–O2	1.950(3)	O1–C1	1.330(4)
C11–C12	1.374(6)	Mo1–O3	1.703(3)	O2–C10	1.336(5)
C12–C13	1.388(6)	Mo1–O4	1.704(2)	O5–N3	1.229(5)
C13–C14	1.367(6)	N1–C1	1.288(5)	O6–N3	1.225(6)
C15–C16	1.385(6)	N1–N2	1.397(5)		
C16–C17	1.398(5)	N2–C8	1.297(5)		
Mo1–N2	2.250(3)	N3–C13	1.466(6)		

Table S3. Hydrogen bond parameters in the crystal structures of prepared complexes $[MoO_2(L)(MeOH)]$ (**1**), $[MoO_2(L)(H_2O)]$ (**2**) and $[(MoO_2(L))_2(4,4\text{-bpy})]$ (**3**)..

$[MoO_2(L)(MeOH)]$ (**1**)					
D–H⋯A	D-H	H⋯A	D⋯A	∠D–H⋯A	Symmetry code
O5–H5⋯N1	0.82(5)	1.97(5)	2.779(4)	171(5)	1–*x*, 1–*y*, 2–*z*
C8–H8⋯O3	0.95	2.52	3.170(4)	126	–*x*, 1–*y*, 2–*z*
$[MoO_2(L)(H_2O)]$ (**2**)					
D–H⋯A	D-H	H⋯A	D⋯A	∠D–H⋯A	Symmetry code
O5–H5A⋯N1	0.88	2.15	2.904(14)	143	1–*x*, 1–*y*, 1–*z*
O5–H5B⋯O6	0.88	2.11	2.971(15)	168	–1+*x*, 1/2–*y*, –1/2+z
C4–H4⋯O4	0.95	2.36	3.208(18)	149	–*x*, 1–*y*, 1–*z*
C7–H7⋯O3	0.95	2.59	3.431(18)	148	1–*x*, 1/2+*y*, 3/2–*z*
C8–H8⋯O7	0.95	2.59	3.402(18)	144	2–*x*, 1/2+*y*, 3/2–*z*
C12–H12⋯O6	0.95	2.51	3.439(16)	167	2–*x*, –1/2+*y*, 3/2–*z*
C14–H14⋯O7	0.95	2.26	3.152(16)	157	2–*x*, 1/2+*y*, 3/2–*z*
$[(MoO_2(L))_2(4,4\text{-bpy})]$ (**3**)					
D–H⋯A	D-H	H⋯A	D⋯A	∠D–H⋯A	Symmetry code
C8–H8⋯O3	0.95	2.53	3.159(5)	124	1–*x*, 1–*y*, 1–*z*
C12–H12⋯O5	0.95	2.30	3.171(5)	153	3/2–*x*, 1/2+*y*, 1/2–*z*
C14–H14⋯O3	0.95	2.55	3.245(5)	130	1–*x*, 1–*y*, 1–*z*

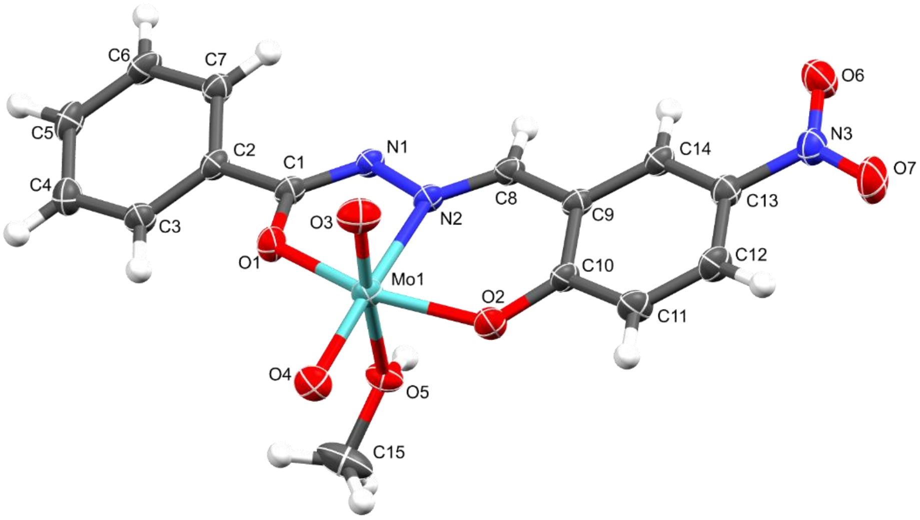

(a)

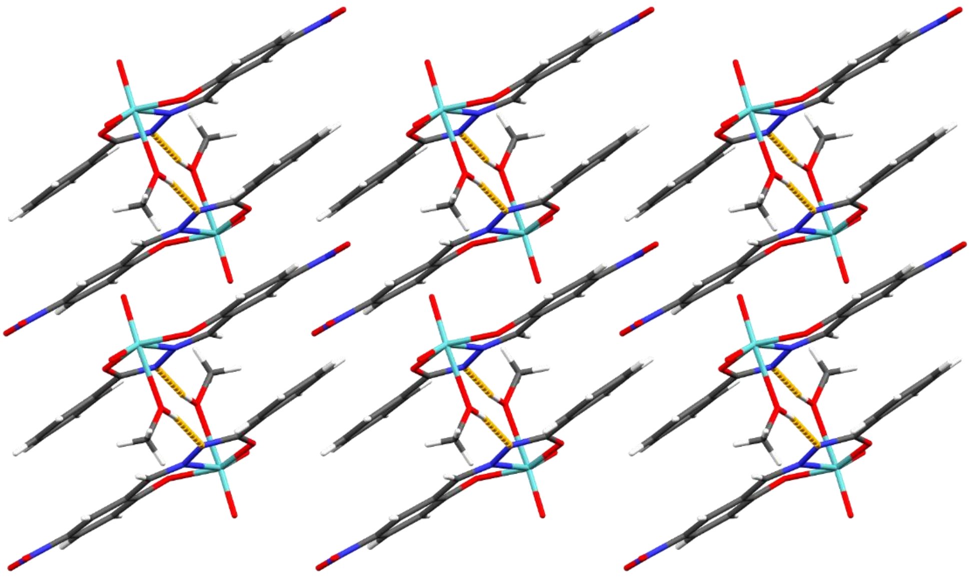

(b)

Figure S9. (a) Molecular structure of $[MoO_2(L)(MeOH)]$ (**1**). Atoms are shown as thermal ellipsoid with 50% probability level. The asymmetric unit contains one whole molecule of the complex. (b) Packing of mononuclear complexes $[MoO_2(L)(MeOH)]$ (**1**) in the crystal structure. The molecules form supramolecular $R_2^2(10)$ homodimers formed by methanol O–H···N1 imido hydrogen bonds. No other significant interactions between the dimers are observed.

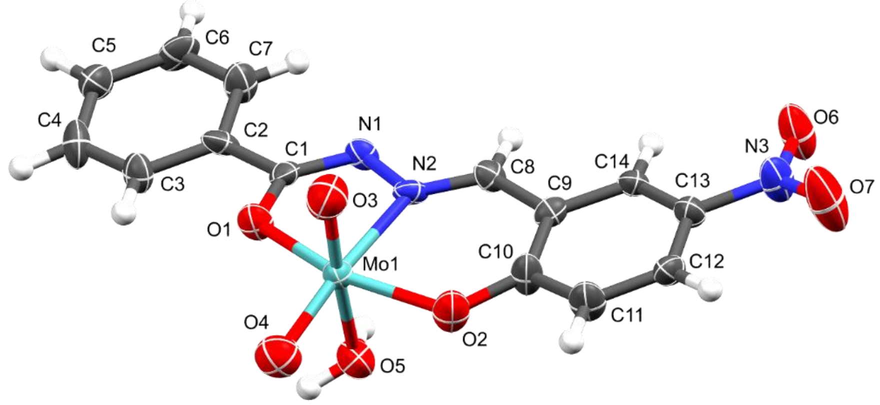
C5
C6
C7
C4
C2
C1
N1
N2
C8
C14
C13
N3
O6
O7
C3
O3
C9
O1
Mo1
C10
C12
O4
O2
C11
O5

(a)

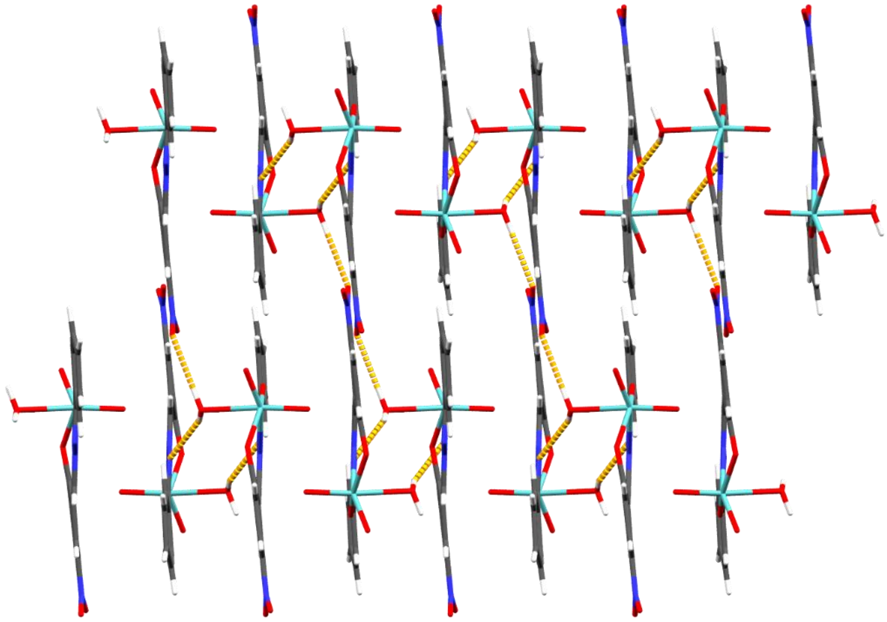

(b)

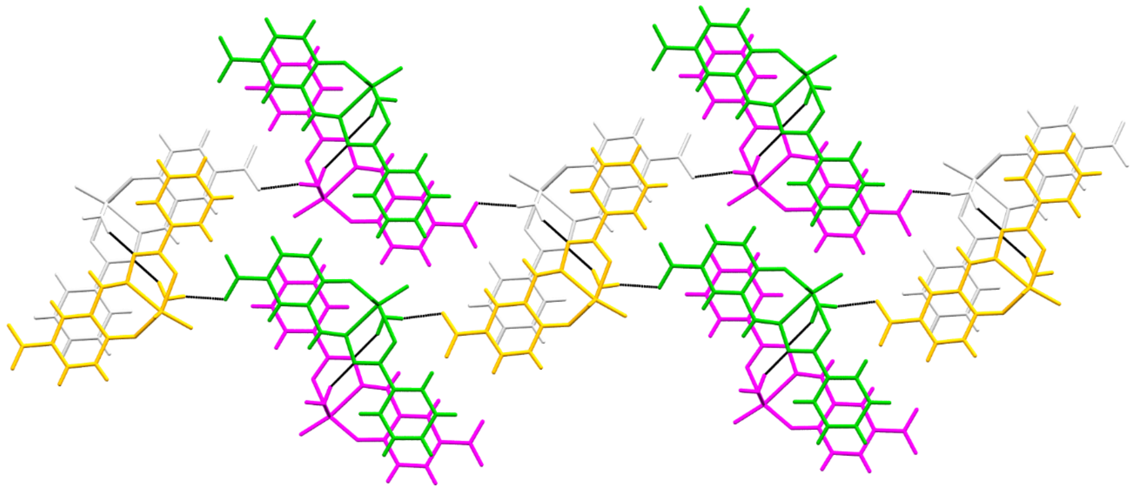

(c)

Figure S10. (a) Molecular structure of $[MoO_2(L)(H_2O)]$ (**2**). Atoms are shown as thermal ellipsoid with a 50% probability level. The asymmetric unit contains one whole molecule of the complex. (b) Packing of mononuclear complexes $[MoO_2(L)(H_2O)]$ (**2**) in the crystal structure. The molecules form supramolecular $C_4^4(26)$ chains spanning through water O–H···O6(nitro) hydrogen bonds and water O–H···N1 imido hydrogen bond. The latter enables the formation of supramolecular homodimers connected via hydrogen bonds with nitro moiety. (c) Packing of mononuclear complexes **2** in the crystal structure. Molecules are colored by symmetry operation, showing the formation of supramolecular dimers bridged by O–H···O6(nitro) hydrogen bonds.

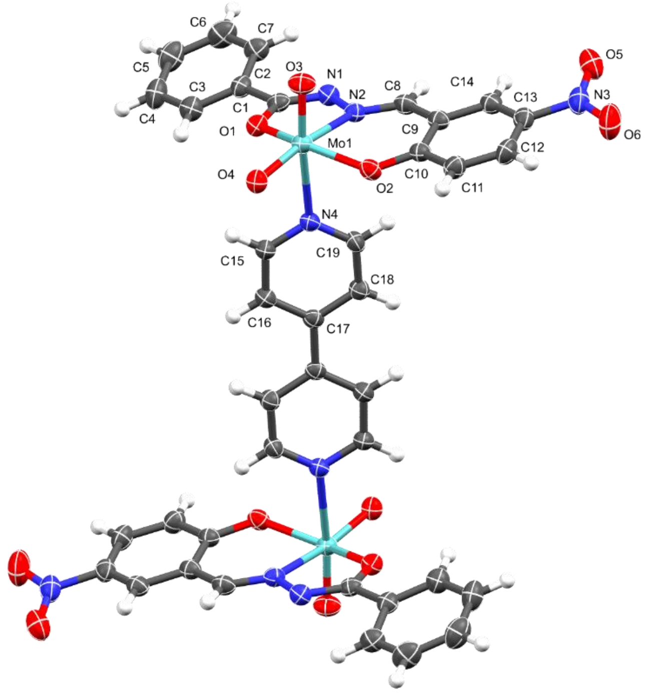

(a)

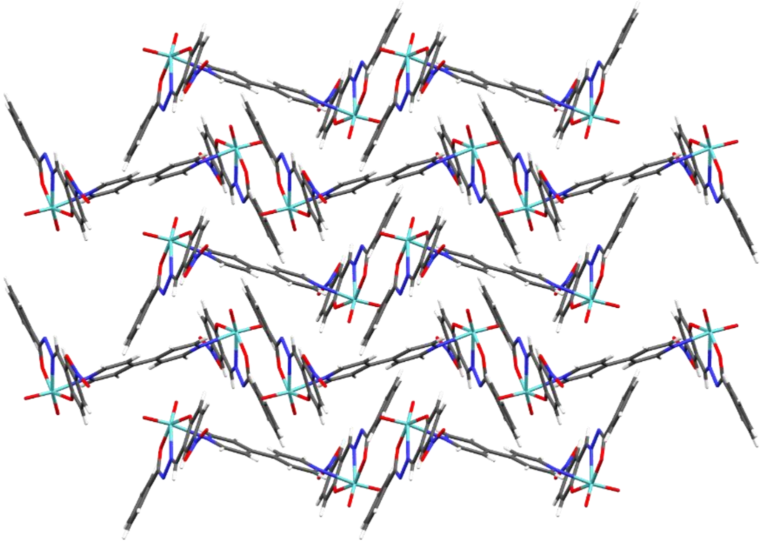

(b)

Figure S11. (a) Molecular structure of [$(MoO_2(L))_2$(4,4-bpy)] (**3**). Atoms are shown as thermal ellipsoid with a 50% probability level. The asymmetric unit contains the upper half of the shown molecule only (center of inversion is situated at the midpoint of 4,4'-bpy C–C bond). (b) Packing of dinuclear complexes [$(MoO_2(L))_2$(4,4-bpy)] (**3**) in the crystal structure. The molecules do not form any significant intermolecular interactions due to the lack of hydrogen bond donors.
